# Ameliorating effect of encapsulated hepatocyte-like cells derived from umbilical cord in high mannuronic alginate scaffolds on acute liver failure in rats

**DOI:** 10.22038/IJBMS.2018.27928.6847

**Published:** 2018-09

**Authors:** Negar Varaa, Saeed Azandeh, Layasadat Khorsandi, Darioush Bijan Nejad, Vahid Bayati, Amin Bahreini

**Affiliations:** 1 Department of Anatomical Sciences, Ahvaz Jundishapur University of Medical Sciences, Ahvaz, Iran; 2 Cellular and Molecular Research Center, Ahvaz Jundishapur University of Medical Sciences, Ahvaz, Iran; 3 Transplantation Ward, Ahvaz Golestan Hospital, Ahvaz Jundishapur University of Medical Sciences, Ahvaz, Iran

**Keywords:** Acetylsalicylic acid, Antioxidants, Epididymis, Melatonin, Sperm, Testosterone

## Abstract

**Objective(s):**

In this study, effects of encapsulated umbilical cord stem cells (UCSCs)-derived hepatocyte-like cells (HLCs) in high mannuronic alginate scaffolds was investigated on CCl_4_-induced acute liver failure (ALF) in rats.

**Material and Methods:**

UCSCs were encapsulated in high mannuronic alginate scaffolds. Then the UCSCs differentiated into HLCs for treatment of CCl_4_-induced ALF in rats. Thirty rats randomly divided into 5 groups: Intoxicated group received only CCl_4_ to induce ALF. In other groups including cell-free, UCSCs and HLCs, alginate scaffolds were transplanted into the liver 4 days after CCl_4_ injection. Biochemical markers including albumin (ALB), blood urea nitrogen (BUN), alanine aminotransferase (ALT), aspartate aminotransferase (AST), and alkaline phosphatase (ALP) were evaluated. Histological changes and gene expression of ALB, alpha-fetoprotein (AFP), and cytokeratin 18 (CK-18) were also assessed.

**Results:**

Expression of CK-18 significantly increased in HLCs compared to the UCSCs in vitro. This indicates that UCSCs can effectively differentiate into the HLCs. In CCl_4_-intoxicated group, BUN, AST and ALT levels, and histological criteria, such as infiltration of inflammatory cells, accumulation of reticulocytes, nuclear pyknosis of hepatocyte and sinusoidal dilation, significantly increased. In this group, ALB secretion significantly decreased, while AFP expression significantly increased. Both UCSCs and HLCs encapsulated in alginate scaffolds effectively attenuated biochemical tests, improved liver cytoarchitecture, increased expression of ALB and reduced AFP expression.

**Conclusion:**

Finding of the present study indicated that encapsulation of UCSCs or HLCs in alginate mannuronic scaffolds effectively improve CCl_4_-induced ALF.

## Introduction

Acute liver failure (ALF) is a rare but sudden clinical syndrome with high rate of mortality, which arises from a fast and extensive hepatocellular necrosis (1). Liver transplantation is the only choice for treatment of ALF. However, scarcity of human donors has strongly limited the transplantation of the liver (2). Currently, regenerative medicine has opened a window for treatment of the end-stage hepatic diseases (3). Tissue engineering has been introduced by using living cells on a suitable substrate or scaffold to restore, maintain, or enhance tissue and organ function (4). 

Mesenchymal stem cells (MSCs) are attractive sources for cell therapy. They can be easily isolated from various tissues including bone marrow, adipose tissue and umbilical cord (5). Umbilical cord mesenchymal stem cells (UCSCs) have unique immunosuppressive properties enabling them to evade host rejection (6).

In liver tissue engineering, in order to MSCs differentiation into mature hepatocytes *in vitro*, there are different methods and growth factors to provide stimuli and maintain cellular function (7). 

A three-dimensional (3D) biocompatible scaffold provides a supporting framework for cell growth, cell-cell or cell-matrix interactions, cell adhesion and cell differentiation (8). 

It has been revealed that 3D culture of hepatocytes keeps their functions better than their culturing in two-dimensional (2D) environment. Aleahmad *et al.* demonstrated that heparin collagen 3D scaffold enhanced hepatocyte differentiation of UCMSCs, and a hepatocyte cell line culture in 3D condition was shown to increase gene expression pattern and metabolic activity compared to 2D environment. 3D culture also improved the expression of hepatic nuclear factor-4, the key regulator of hepatocyte differentiation, in hepatocytes-like cells (HLCs) derived from human Wharton’s jelly MSCs (WJ-MSC). This evidence demonstrated that 3D culture can enhance the hepatocyte function loss and improve the hepatocyte differentiation efficiency (9).

Alginate has been considered as a suitable scaffold that plays a critical role in tissue engineering and enhances the function of mature hepatocytes and maintains mature hepatocyte function within bio-artificial livers (10). Alginate is an unbranched natural copolymer composed of D-mannuronate and L-guluronate, linked together by 1, 4 bonds. Due to its relative stability, biocompatibility, adjustable porosity and simplicity of use, alginate is thus a biomaterial of choice to cell therapy (11).

It has been demonstrated that high mannuronic alginate effectively enhanced albumin (ALB) secretion in encapsulated hepatocytes compared to the high guluronic content encapsulation (12).

Until now, the ideal marker to trace the pathway of stem cell development does not exist, but under all circumstances, alpha-fetoprotein (AFP) is a very promising candidate for the study of differentiation or retrodifferentiation by virtue of its strong correlation with fetal gene expression. In addition, AFP is a biological marker, which increases in liver injuries (13). 

The expression of ALB that is the abundant protein synthesized by mature hepatocytes starts in early fetal hepatocytes and reaches the maximal level in adult hepatocytes. Cytokeratin 18 (CK-18), a cytoskeletal protein, is expressed in mature hepatocytes. Hence, expression level of these genes can clarify the statue of liver function (10).

Several protocols for generating functional hepatocytes have been reported in literature; however, reproducible and efficient differentiation remained challenging under conventional (2D) culture. In this study, we used a 3D culture condition for generating functional HLCs from UCSCs.

Most of the transplantation sites, such as intraperitoneal or subcutaneous, are unable to maintain cell viability for long time periods as the transplanted cells have poor access to oxygen and nutrients (14). The present study was conducted to investigate whether the transplantation of mannuronic-rich alginate beads encapsulated UCSCs-derived HLCs into the liver could ameliorate ALF induced by carbon tetrachloride (CCl_4_) in rats while cell-scaffold constructs were implanted directly into the liver between the lobes.

## Materials and Methods

***Isolation of UCSCs***

Fresh human umbilical cords were collected aseptically from the mothers undergoing uncomplicated elective cesarean section after receiving their written consent. The umbilical cords dissected to earn small pieces of Wartons’ jelly. The Wartons’ jelly pieces were cultured as explants in DMEM (low glucose) with 20% fetal bovine serum. 

The explants were left undisturbed for 3 days in CO_2_ incubator at 37 ^°^C. After 10 days, the explants were removed from flasks. After 5 days, the flasks were passaged and the isolated cells were used in the experiment (15). The isolated cells were also characterized using flowcytometry and assessment of differentiation potential.

***UCSCs characterization***

After trypsinization, 5 × 10^5^ cells were resuspended in PBS and 5% FBS and incubated with phycoerythrin (PE)- or fluorescein isothiocyanate (FITC)-conjugated antibodies against mesenchymal cell surface markers, CD73 and hematopoietic markers, CD31 and IgG (ebio science, USA) for 30 min at 4°C. Labeled cells were assayed by Dako Glaxy flowcytometer. Mouse isotype-matched antibody was used as controls (ebio science, USA).

UCSCs were differentiated toward the adipogenic and osteogenic linage according to the protocol described by Hashemitabar *et al* (15).

***Encapsulation of UCSCs and hepatic differentiation***

A low viscosity, high mannuronic acid alginate solution (2% wt/vol, Nova Matrix, Philadelphia, PA) was prepared. Briefly, the alginate powder was dissolved in buffer solution containing 0.15 M NaCl and 0.025 M HEPES (4-(2-hydroxyethyl)-1-piperazineethanesulfonic acid) (16). The isolated UCSCs at a density of 3×10^6^ cells/ml were resuspended in 3 ml of the aforementioned solution and dropped into a 10^2^ mM CaCl_2_ solution and allowed to be polymerized for 20 min. Then, alginate scaffolds containing UCSCs were rinsed 3 times in 0.15 M NaCl. The constructs were finally cultured in 25-cm^2^ T-flasks. Differentiation of UCSCs into HLCs was induced using a 4-step protocol as follows (17):

Step I: DMEM (low glucose) and 10 ng/ml fibroblast growth factor (FGF-4) for 2 days.

Step II: DMEM (low glucose), 1% insulin-transferrin-selenium (ITS), and 20 ng/ml hepatocyte growth factor (HGF) for 2 days. 

Step III: DMEM (low glucose), 100 nM dexamethasone, 1% ITS, 10^−7^ mg glucagon, 10 ng/ml oncostatin M (OSM) and 20 ng/ml HGF for 2 days. 

Step IV: Step III medium and 100 nM trichostatin A (TSA) or 1% dimethyl sulfoxide (DMSO) for 14 days. The medium was exchanged every 2 days (17).

***Scanning electron microscopy***

The alginate free-cell scaffolds, UCSCs scaffolds and HLCs scaffolds were assessed by a scanning electron microscope (SEM) before transplantation. Briefly, the scaffolds on day 14 were washed twice with cold PBS and fixed by 2.5% glutaraldehyde for 24 hr. The scaffolds were incubated with 2.5% glutaraldehyde buffered in 0.15 mol/l sodium cacodylate at 20˚C for 1 hr (pH=7.2). After fixation, the scaffolds were repeatedly rinsed in cacodylate buffer. The cultures were then dehydrated in a graded series of ethanol (50, 70, 95 and 100% alcohol). Then, chemical drying was achieved by using ethanol/Hexamethyldisilazane (HMDS, Merck, Germany) mixture and pure HMDS. The preparations were sputter-coated with gold-palladium before SEM (18). Porosity of the scaffolds was determined by the free ImageJ software. (http://imagej.nih.gov/ij/index.html)

***Animals***

The study protocol was approved by the Ethics Committee of Ahvaz Jundishapour University of Medical Sciences (approve number: IR. Ajums. REC. 1394. 605). 

 Thirty rats (Albino, male, 250 to 300 g) were obtained from Experimental Animals Research Center of Ahvaz Jundishapur University of Medical Sciences and housed for 1 week in the animal room of the Department of Anatomical Sciences for adaptation to the environmental before the experiments. The experimental room had a controlled temperature at 20± 5 ^°^C with 12 hr light/dark cycles. The animals had access to food and water freely. 

***Liver surgery***

All surgeries were performed under deep anesthesia and sterile surgical techniques. Animals were anesthetized with 10% ketamine and 2% xylazine (Alfasan, Woerden, Netherlands) through intraperitoneal injection and then fixed in supine position. The surgical area was disinfected with iodophore. A midline abdominal incision (2 to 3 cm) was made and alginate scaffolds were placed between the lobes of the liver (The 15 alginate sheets, with a diameter 6 mm and height 1 mm). Then, the abdominal incision was sutured in two layers with 5-0 absorbable suture. Each rat was maintained in a separate cage and checked daily for their health status. To induce ALF, CCl_4_ at the dose of 400 μl/kg was peritonealy injected for two consecutive days. CCl_4_ was dissolved in olive oil (1:1) (19). 

***Experimental design***

The rats were randomly divided into 5 groups:

1- Negative control group: received olive oil via intrapritoneal injection. 

2- CCl_4_-intoxicated group: received only CCl_4_ via intrapritoneal injection. 

3- Cell-free alginate transplanted group: cell-free scaffolds were transplanted into the liver 4 days after administration of CCl_4_.

4- UCSCs transplanted group: UCSCs alginate scaffolds equivalent to one million cells were transplanted into the liver 4 days after administration of CCl_4_.

5- HLCs alginate beads transplanted group: HLCs alginate scaffolds equivalent to one million cells were transplanted into the liver 4 days after administration of CCl_4_.

Fourteen days after transplantation, the rats were euthanized, the liver tissues removed and fixed in 10% neutral formalin solution for histological assessments (20).

***Biochemical tests***

Blood samples were collected and centrifuged for 15 min at 10,000 g before and after the beginning of the experiments. The supernatant was stored at -80 ^°^C until use. ALB, blood urea nitrogen (BUN), alanine aminotransferase (ALT), aspartate aminotransferase (AST), and alkaline phosphatase (ALP) were determined using photometric kits (Pars Azmun, Tehran, Iran) according to the manufacturer’s instruction.

***Histological evaluation***

After blood collection, the rat livers were fixed in 10% formalin solution. Paraffin sections of 4-6 µm were prepared and stained with hematoxylin and eosin (H&E). Six microscopy slides per animal were examined for assessment of histological changes such as congestion of erythrocytes, infiltration of inflammatory cells, nuclear pyknosis and sinusoidal dilation. For assessment of nuclear pyknosis, the average percentage of nuclear pyknosis was determined by dividing the number of nuclei with the pyknosis in a randomly microscopic field by the total number of nuclei in the same field and the result multiplied by 100. Infiltration of inflammatory cells, sinusoidal dilation and congestion of RBCs were graded into 4 categories: normal (0), weak (1), moderate (2) or intense (3) and the averages were considered. For each slide, the mean of 6 fields was calculated. Slides were read in a “blind” fashion (21).

***Real-time RT-PCR analysis***

Real-time RT-PCR was performed on day 14 after transplantation to determine the mRNA expression levels of ALB, AFP, and CK-18*. *The RNA was isolated from the liver tissue by using the RNeasy plus Mini Kit (Qiagen, Gaithersburg, MD, USA) according to the manufacturer’s instructions. The extracted RNAs were quantified using a nanodrop spectrophotometer (Thermo Scientific NanoDrop 2000c Spectrophotometer, USA). 

The cDNA was synthesized using a QuantiTect Reverse Transcription Kit (Qiagen, Gaithersburg, MD, USA).

The primer sequences used in this study were ALB: 5-GCCTGCTGACTTGCCTTCATTAG-3 (forward primer) and 5- TCAGCAGCAGCACGACAGAGTA-3 (reverse primer);

AFP: 5-GAAACCCACTGGAGATGAACAGTC-3 (forward primer) and 5-AAGTGGGATCGATGCAGGA-3 (reverse primer);

CK-18: 5-GATCGACCTGGACTCCATGAGAA-3 (forward primer) and 5-CCGTTGAGCTGCTCCATCTGTA-3 (reverse primer);

GAPDH: were 5-TGCTGGTGCTGAGTATGTCGTG-3 (forward primer) and 5-CGGAGATGATGACCCTTTTGG-3 (reverse primer). 

PCR amplification was performed over 40 cycles using a SYBR Green PCR Master Mix (Takara, Beijing, china) with the following program: 95 ˚C for 10 min, 95 ˚C for 15 sec, 5 ˚C for 45 sec and 60 ˚C for 40 sec. Data were analyzed using the 2^-ΔΔCT^ method. Expression values were corrected for the housekeeping genes glyceraldehyde-3-phosphate dehydrogenase (GAPDH) (22). 

***Statistical analysis***

Data were presented as mean ± standard deviation and were statistically analyzed using SPSS software (Version 22, Chicago, Illinois, USA) by one-way analysis of variance (ANOVA). The difference between the means was considered significant when *P* was < 0.05.

## Results

***UCSCs characterization***

Flowcytometric analysis indicated high expression level of CD 73 (88.9%), while low expression of CD31 (0.12%) was observed in the UCSCs.

**Figure 1:**
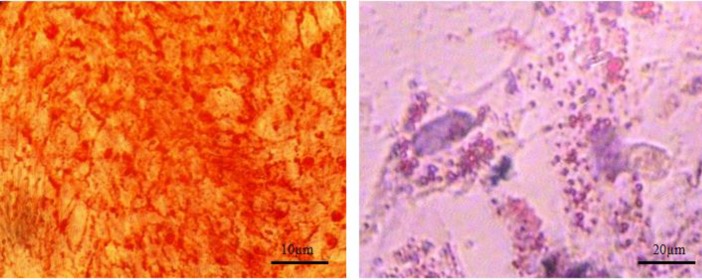
Differentiation of UCSC into adipocytes (right) and osteoblasts (left) have been shown

The cells treated with adipogenic induction medium for 21 days showed numerous lipid vacuoles by using Oil Red O staining. In osteogenic induction medium, deposition of calcium showed red color by Alizarin Red stating. In non-treated control cells, no mineralized matrix and lipid vacuole were observed (Figure 1).

**Table 1:** Histological criteria in control and experimental groups

Histological criteria	Groups				
	Control	CCl_4_	Cell-free beads	UCSCs beads	HLCs beads
Congestion of RBC	0.0 ± 0.0	2.5 ± 0.3*	2.8 ± 0.12*	0.41 ± 0.4*##	0.35 ± 0.04*##
Infiltration of inflammatory cells	0.01 ± 0.001	1.8 ± 4.16*	2.1 ± 4.3*	0.43 ± 0.05*##	0.2 ± 0.09*##
Sinusoidal dilatation	0.04 ± 0.002	2.48 ± 0.25*	2.53 ± 0.19*	1.4 ± 0.05*#	1.35 ± 0.07*#
Pyknosis (%)	0.01 ± 0.002	36.3 ± 5.3*	38.3 ± 3.8	13.9 ± 2.6*##	12.3 ± 2.9*##

Values are expressed as mean ± SD.

* and # symbols respectively indicate comparison to the control and CCl_4_-intoxicated rats groups. UCSCs: Umbilical cord stem cells, HLCs: Hepatocyte-like cells.

* *P *< 0.001,

# *P *< 0.05,

## *P *< 0.01;

**Figure 2:**
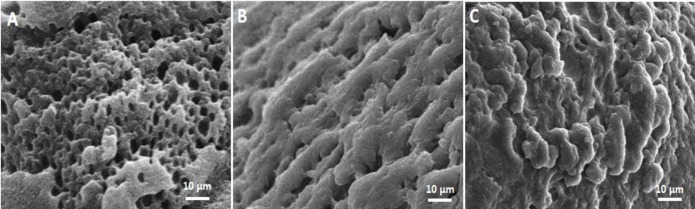
Photomicrograph of scanning electron microscope (SEM) staining. A, cell-free alginate bead; B, umbilical cord stem cells (UCSCs) alginate bead; C, hepatocyte-like cells (HLCs) alginate bead

**Figure 3:**
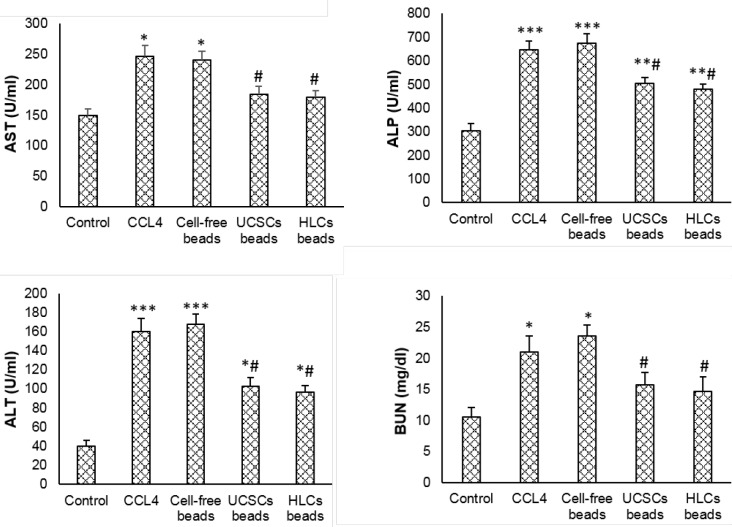
Plasma levels of biochemical markers in control and experimental treated groups. Values expressed as mean±SD for 6 rats. *P<0.05, **P<0.01, ***P<0.001, # *P*<0.05; * and # symbols respectively indicate comparison to the control and CCl_4_-intoxicated groups. UCSCs: Umbilical cord stem cells, HLCs: Hepatocyte-like cells

**Figure 4:**
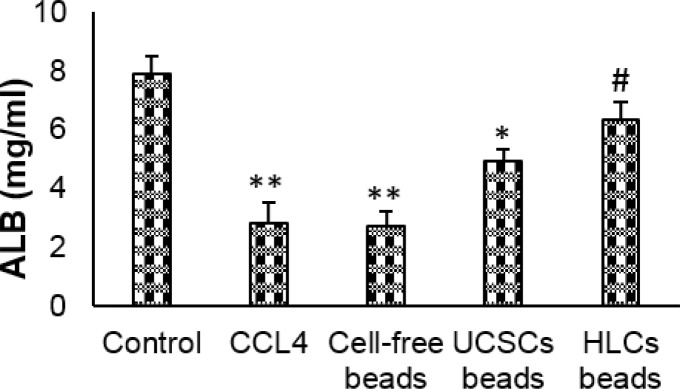
Albumin (ALB) secretion in control and experimental treated groups. Values expressed as mean±SD for 6 rats. *P<0.05, **P<0.01, # *P*<0.05; * and # symbols respectively indicate comparison to the control and CCl_4_-intoxicated groups. UCSCs: Umbilical cord stem cells, HLCs: Hepatocyte-like cells

**Figure 5:**
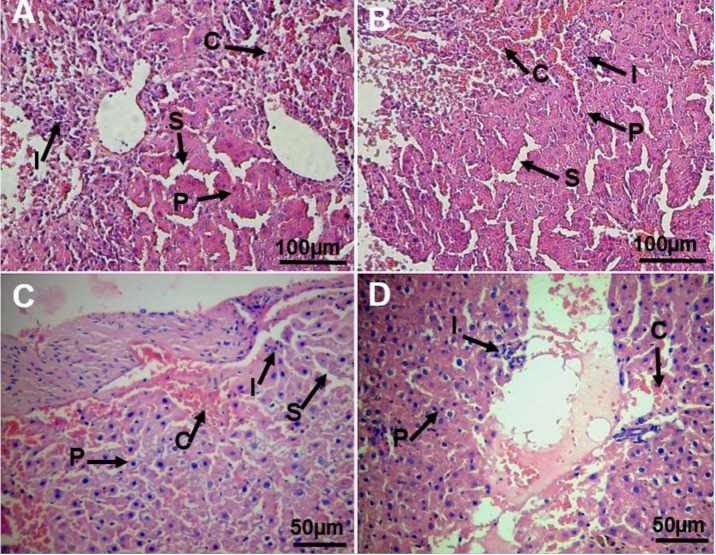
Light microscopy of cross sections of H&E stained liver tissue. A: CCl_4_ group; B: cell-free beads transplanted group; C: Umbilical cord stem cells (UCSCs) beads transplanted group; D: Hepatocyte-like cells (HLCs) beads transplanted group. C: congestion of RBCs, I: infiltration of inflammatory cells, P: nuclear pyknosis, S: sinusoidal dilation

**Figure 6:**
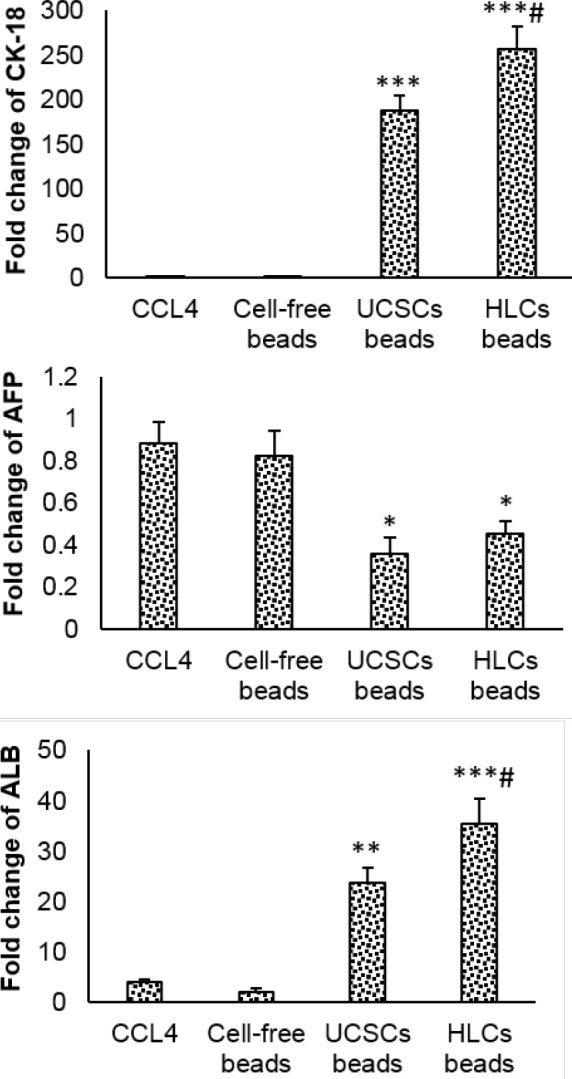
Gene expression in treated groups. Values expressed as mean±SD for 6 rats. *P<0.05, **P<0.01, ***P<0.001, # *P*<0.05; * and # symbols respectively indicate comparison to the control and CCl_4_-intoxicated groups. UCSCs: Umbilical cord stem cells, HLCs: Hepatocyte-like cells

***Morphology assessments***

UCSCs had fibroblast like shape. HLCs were close together and formed cell clusters. Inverted microscopy reveled cell clusters that we call them hepatospher, since they had clear cytoplasm and nuclei.

In the SEM images, the cell-free porous scaffolds had numerous interconnected pores. The scaffolds had porosity with mean pore size of 176.88±0.25 SEM evaluations showed that UCSCs and HLCs formed continuous sheets of cells. The morphology of encapsulated UCSCs was flat and elongated. While, the encapsulated HLCs showed polyhedral morphology with confluence similar to the hepatic-like clusters (Figure 2).

***Biochemical tests***

In CCl_4_-intoxicated group, AST, ALT, ALP and BUN levels significantly increased and ALB concentration significantly decreased compared to negative control and both cell-treated groups (*P*<0.05). Transplantation of cell-free scaffolds had no influence on the concentration of the biochemical markers. Transplantation of both encapsulated UCSCs and HLC induced a significant decrease in AST, ALT, ALP and BUN levels and significantly increased ALB secretion (*P*<0.05). The levels of the biomarkers and ALB were more pronounced in HLC transplantation in comparison with the UCSCs transplanted animals (*P*<0.05). (Figures 3 and 4).

***Histology***

Normal liver architecture was observed in control group. Lobular structures of the liver were damaged in CCl_4_-intoxicated rats. Accumulation of erythrocytes, infiltration of inflammatory cells, nuclear pyknosis and sinusoidal dilation were observed in this group. In cell-free scaffold transplanted animals, histological criteria were similar to those in the CCl_4_-intoxicated rats. The histological criteria significantly improved in both UCSCs and HLCs transplanted-animals in comparison with the CCl_4_-treated rats. The liver histology was the same in the UCSCs and HLCs transplanted-rats (Figure 5 and Table 1).

***Gene expression ***

In cell-free scaffold transplanted group, expression of ALB, AFP and CK-18 was similar to the CCl_4_-treated animals. In both UCSC and HLC transplanted-groups, ALB and CK-18 expression significantly increased, while AFP expression significantly decreased compared to the CCl_4_-intoxicated rats. There was no significant differentiation in expression of AFP between UCSCs and HLCs transplanted groups. Expression of ALB and CK-18 genes in HLCs transplanted-group significantly increased compared to the UCSCs-transplanted animals (Figure 6).

## Discussion

The results of this study demonstrated that administration of both UCSCs and HLCs improve the liver functions. Hu *et al.* (2015) have reported that somatic stem cells could effectively differentiate in alginate micro beads into HLCs and secret ALB (23). The HLCs encapsulated in alginate scaffolds alleviated ALF induced by CCl_4_ when transplanted into the liver of rats. In a study, injection of UCSCs-derived HLCs effectively improved CCl_4_–induced liver damage in mice (24). Transplantation of the encapsulated hepatocytes in alginate/poly-L-lysine improved the hepatic function in a 90% partial hepatectomy of rats (14). Therefore, cell therapy could ameliorate CCL_4_-incuced hepatotoxicity.

In this study, ALT and AST levels that are important indicators of liver injury were decreased in UCSC and HLC-treated animals. The serum activity of these enzymes is routinely measured for the detection of liver disorders (25). The liver cells release these enzymes into the blood stream following injury or death. ALP levels also decreased in treated animals. In acute hepatitis, presenting cholestasis and peripheral arterial disease, ALP usually remains normal or moderately increased (25). Reversal of the elevation of plasma enzyme activity in CCl_4_-induced hepatotoxicity indicates that the transplanted cells may decrease liver cell death.

AFP significantly decreased in both UCSCs and HLCs transplanted-rats. In addition, ALB secretion significantly increased after transplantation of these cells. This indicates that both UCSCs and HLCs have hepato-protective effects.

The results of the histological study also support the results of biochemical parameters. Transplantation of HLCs effectively attenuated histological changes such as nuclear pyknosis, sinusoidal dilation, congestion of RBCs, and infiltration of inflammatory cells compared to the rats treated with CCl_4_ alone.

The alteration in ALB secretion and gene expression was accompanied by histological improvement in both UCSC and HCLs transplantation. Transplantation of hepatic-differentiated human amniotic epithelial cells in a mice cirrhosis model led to a successful *in vivo *engraftment and differentiation into functional hepatocytes (26). 

The present study showed that the encapsulated UCSCs also attenuated ALF when transplanted into the liver. Li *et al.* (2014) showed that hydrogel scaffolds loaded with UCSCs significantly improved liver damage (8). It has been shown that intraportal transplantation of human bone marrow MSCs prevents hepatocellular degeneration induced by D-galactosamine in pigs (27). Findings have also shown that fluorescently labeled MSCs can accumulate in liver and alleviate CCl_4_-induced liver damage in mice (28). In agreement with our findings, Zhou *et al.* (2014) have demonstrated that UCSCs and UCSCs-derived hepatocytes have similar hepato-protective effect in an ALF mice model (29). 

Although both UCSC and HLC transplantation ameliorated the adverse impact of CCl_4_, the biochemical tests revealed a significant reduction of ALB secretion in UCSC-treated animals compared to the HLCs-transplanted animals. 

Transplantation of human induced pluripotent stem cells -derived HLCs attenuated ALT, AST and BUN levels in ALF-induced D-galactose-treated rats. These cells also markedly increased ALB secretion in D-galactose-treated rats (30) and CCl_4_ treated-mouse (31). Injection of UCSCs and adipose stem cells via the tail vein improved liver failure, and decreased ALT and AST levels in CCl_4_-treated mice (32, 33).

In the present study, UCSCs in alginate beads expressed hepatocyte-specific markers (CK-18 and ALB) after two weeks of transplantation in CCl_4_-treated rats. However, we could not clearly prove that the transplanted UCSCs were fully differentiated into cells with functions comparable to endogenous hepatocytes.

In the current study, it was found that transplantation of UCSCs markedly improved liver functions and also increased ALB, AFP and CK18 expression level. This finding indicates that the UCSCs may differentiate into the HLCs in liver niche or produce cytokines that act as chemoattractant for hepatocyte progenitor cells. It is also possible that stimulating factors such as complement system, platelets, inflammatory cytokines, growth and anti-inflammatory factors (34) that are secreted from damaged liver tissue affect UCSCs and induce differentiation of these cells into HLCs. Besides, it has been reported that 3D culture system influences the liver-specific marker expression pattern in the absence or presence of chemical induction (35). Therefore, 3D culturing may also promote the differentiation toward hepatocytes. However, our methodology did not support these declarations.

We showed the anti-inflammatory effects of both HLCs and UCSCs on ALF liver according to scoring system. There are some reports that also confirm the anti-inflammatory effects of both USCSs and HLCs after transplantation. (36). Tuo *et al.* (2017) showed that UCSCs can inhibit immune response, promote hepatocyte regeneration, alleviate the progression of liver fibrosis, and improve liver function in CCl_4_-induced hepatotoxicity (37).

Tumor formation is the most important problem of cell therapy. Tumorigenicity is a common problem of embryonic stem cells or induced pleuripotent stem cells transplantations. However, tumorigenicity of MSCs is very low (38). It was previously reported that differentiated MSCs were more stable and safer than undifferentiated MSCs (7). 

There are many transplantation sites, including omentum, small intestinal mesentery, and subcutaneous space of the abdominal wall (39). In most studies, the intraperitoneal cavity is used as the site of transplantation, which may cause fibrosis around the encapsulated cells (14). Transplantation of the adipose-derived stem cells in the spleen, as another transplantation site, has been reported to fail the delivery of the cell to the liver (40). This study suggests that the insertion of cells in the alginate scaffolds into the liver is better than other sites.

## Conclusion

This study demonstrated that transplantation of UCSCs and HLCs in alginate scaffolds improve CCl_4_-induced ALF. It seems that USSCs is as good as HLCs in curing ALF. However, the mechanisms such as paracrine, anti-inflammatory, and antiapoptotic effects of transplanted UCSCs and HLCs in alginate beads remain to be resolved.

## References

[1] Sun K, Xie X, Xie J, Jiao S, Chen X, Zhao X (2014). Cell-based therapy for acute and chronic liver failures: distinct diseases, different choices. Sci Rep.

[2] Bernal W, Auzinger G, Dhawan A, Wendon J (2010). Acute liver failure. Lancet.

[3] Russo FP, Parola M (2012). Stem cells in liver failure. Best Pract Res Clin Gastroenterol.

[4] Sipe JD (2002). Tissue engineering and reparative medicine. Ann N Y Acad of Sci.

[5] Zhao Q, Ren H, Zhu D, Han Z (2009). Stem/progenitor cells in liver injury repair and regeneration. Biol Cell.

[6] Fu YS, Cheng YC, Lin MYA, Cheng H, Chu PM, Chou SC (2006). Conversion of human umbilical cord mesenchymal stem cells in Wharton’s jelly to dopaminergic neurons in vitro: potential therapeutic application for Parkinsonism. Stem cells.

[7] Wu XB, Tao R (2012). Hepatocyte differentiation of mesenchymal stem cells. Hepatobiliary Pancreat Dis Int.

[8] Li P, Zhang J, Liu J, Ma H, Liu J, Lie P (2014). Promoting the recovery of injured liver with poly (3-hydroxybutyrate-co-3-hydroxyvalerate-co-3-hydroxyhexanoate) scaffolds loaded with umbilical cord-derived mesenchymal stem cells. Tissue Eng Part A.

[9] Aleahmad F, Ebrahimi S, Salmannezhad M, Azarnia M, Jaberipour M, Hoseini M (2017). Heparin/collagen 3D scaffold accelerates hepatocyte differentiation of Wharton’s jelly-derived mesenchymal stem cells. Tissue Eng Regen Med.

[10] Fang S, Qiu Yd, Mao L, Shi Xl, Yu DC, Ding Yt (2007). Differentiation of embryoid-body cells derived from embryonic stem cells into hepatocytes in alginate microbeads in vitro. Acta Pharmacol Sin.

[11] Gautier A, Carpentier B, Dufresne M, Vu Dinh Q, Paullier P, Legallais C (2011). Impact of alginate type and bead diameter on mass transfers and the metabolic activities of encapsulated C3A cells in bioartificial liver applications. Eur Cell Mater.

[12] Ka halil M, Shariat Panahi A, Tootle R, Ryder T, Mc Closkey P, Roberts E (2001). Human hepatocyte cell lines proliferating as cohesive spheroid colonies in alginate markedly upregulate both synthetic and detoxificatory liver function. J Hepatolo.

[13] Kuhlmann WD, Peschke P (2006). Hepatic progenitor cells, stem cells, and AFP expression in models of liver injury. Int J Exp Pathol.

[14] Aoki T, Jin Z, Nishino N, Kato H, Shimizu Y, Niiya T (2005). Intrasplenic transplantation of encapsulated hepatocytes decreases mortality and improves liver functions in fulminant hepatic failure from 90% partial hepatectomy in rats. Transplantation.

[15] Hashemitabar M, Allahbakhshi E, Tabande MR, Orazizadeh M, Dehbashi FN, Azandeh S (2015). Isolation and characterization of human umbilical cord mesenchymal stem cells and their differentiation into Pdx-1+ Cells. Int J Biomed Sci.

[16] Chen MJ, Lu Y, Simpson NE, Beveridge MJ, Elshikha AS, Akbar MA (2015). In situ transplantation of alginate bioencapsulated adipose tissues derived stem cells (ADSCs) via hepatic injection in a mouse model. PLos One.

[17] Yoon HH, Jung BY, Seo YK, Song KY, Park JK (2010). In vitro hepatic differentiation of umbilical cord-derived mesenchymal stem cell. Process Biochem.

[18] Soleimani M, Khorsandi L, Atashi A, Nejaddehbashi F (2014). Chondrogenic differentiation of human umbilical cord blood-derived unrestricted somatic stem cells on A 3D beta-tricalcium phosphate-alginate-gelatin scaffold. Cell J.

[19] Saif A, Sarhan O, Mohamed Elmogy M, Mutwally H (2014). Hepatoprotective effects of Zamzam water against Carbon Tetrachloride induced liver damage in Rats: Biochemical, Histopathological and molecular evidences. Life Sci.

[20] Suvarna KS, Layton C, Bancroft JD (2018). Bancroft’s Theory and Practice of Histological Techniques E-Book.

[21] Ishak K, Baptista A, Bianchi L, Callea F, De Groote J, Gudat F (1995). Histological grading and staging of chronic hepatitis. J hepatol.

[22] Azandeh S, Gharravi AM, Orazizadeh M, Khodadi A, Hashemi Tabar M (2016). Improvement of mesenchymal stem cell differentiation into the endoderm lineage by four step sequential method in biocompatible biomaterial. Bioimpacts.

[23] Hu C, Li L (2015). In vitro and in vivo hepatic differentiation of adult somatic stem cells and extraembryonic stem cells for treating end stage liver diseases. Stem Cells Int.

[24] Seo MJ, Suh SY, Bae YC, Jung JS (2005). Differentiation of human adipose stromal cells into hepatic lineage in vitro and in vivo. Biochem Biophys Res Commun.

[25] Gowda S, Desai PB, Hull VV, Math AAK, Vernekar SN, Kulkarni SS (2009). A review on laboratory liver function tests. Pan Afr Med J.

[26] Lin JS, Zhou L, Sagayaraj A, Jumat NHB, Choolani M, Chan JKY (2015). Hepatic differentiation of human amniotic epithelial cells and in vivo therapeutic effect on animal model of cirrhosis. J Gastroenterol Hepatol.

[27] Li J, Zhang L, Xin J, Jiang L, Zhang T, Jin L (2012). Immediate intraportal transplantation of human bone marrow mesenchymal stem cells prevents death from fulminant hepatic failure in pigs. Hepatology.

[28] Aldridge V, Garg A, Davies N, Bartlett DC, Youster J, Beard H (2012). Human mesenchymal stem cells are recruited to injured liver in a β1-integrin and CD44 dependent manner. Hepatology.

[29] Zhou R, Li Z, He C, Li R, Xia H, Li C (2014). Human umbilical cord mesenchymal stem cells and derived hepatocyte-like cells exhibit similar therapeutic effects on an acute liver failure mouse model. PLos One.

[30] Ramanathan R, Pettinato G, Beeston JT, Lee DD, WenX, Mangino MJ (2015). Transplantation of human stem cell-derived hepatocytes in an animal model of acute liver failure. Surgery.

[31] Asgari S, Moslem M, Bagheri-Lankarani K, Pournasr B, Miryounesi M, Baharvand H (2013). Differentiation and transplantation of human induced pluripotent stem cell-derived hepatocyte-like cells. Stem Cell Rev.

[32] Elmahdy NA, Sokar SS, Salem ML, Sarhan NI, Abou Elela SH (2017). Anti-fibrotic potential of human umbilical cord mononuclear cells and mouse bone marrow cells in CCl4-induced liver fibrosis in mice. Biomed Pharmacother.

[33] Deng L, Liu G, Wu X, Wang Y, Tong M, Liu B (2014). Adipose derived mesenchymal stem cells efficiently rescue carbon tetrachloride-induced acute liver failure in mouse. Sci World J.

[34] Cienfuegos J, Rotellar F, Baixauli J, Martínez Regueira F, Pardo F, Hernández Lizoáin JL (2014). Liver regeneration the best kept secret, A model of tissue injury response. Rev Esp Enferm Dig.

[35] Khodabandeh Z, Vojdani Z, Talaei-Khozani T, Jaberipour M, Hosseini A, Bahmanpour S (2016). Comparison of the expression of hepatic genes by human wharton’s jelly mesenchymal stem cells cultured in 2D and 3D collagen culture systems. Iran J Med Sci.

[36] Burra P, Arcidiacono D, Bizzaro D, Chioato T, Di Liddo R, Banerjee A (2012). Systemic administration of a novel human umbilical cord mesenchymal stem cells population accelerates the resolution of acute liver injury. BMC gastroenterol.

[37] Tuo L, Zeng W, Xue H, Wu X (2017). Umbilical cord mesenchymal stem cells and their association with liver fibrosis. Zhonghua Gan Zang Bing Za Zhi.

[38] Liu Z, Meng F, Li C, Zhou X, Zeng X, He Y (2014). Human umbilical cord mesenchymal stromal cells rescue mice from acetaminophen-induced acute liver failure. Cytotherapy.

[39] Umehara Y, Hakamada K, Seino K, Aoki K, Toyoki Y, Sasaki M (2001). Improved survival and ammonia metabolism by intraperitoneal transplantation of microencapsulated hepatocytes in totally hepatectomized rats. Surgery.

[40] Chen YX, Zeng ZC, Sun J, Zeng HY, Huang Y, Zhang ZY (2015). Mesenchymal stem cell–conditioned medium prevents radiation-induced liver injury by inhibiting inflammation and protecting sinusoidal endothelial cells. J Radiat Res.

